# Increasing accuracy of pulse transit time measurements by automated elimination of distorted photoplethysmography waves

**DOI:** 10.1007/s11517-017-1642-x

**Published:** 2017-03-30

**Authors:** Marit H. N. van Velzen, Arjo J. Loeve, Sjoerd P. Niehof, Egbert G. Mik

**Affiliations:** 1 Department of Anesthesiology, Laboratory of Experimental Anesthesiology, Erasmus University Medical Center, Room Ee2381, PO Box 2040, 3000 CA Rotterdam, The Netherlands; 2 Department of BioMechanical Engineering, Faculty 3mE, Delft University of Technology, Delft, The Netherlands

**Keywords:** Photoplethysmography (PPG), Pressure pulse waves (PWs), Pulse transit time (PTT), Algorithm

## Abstract

Photoplethysmography (PPG) is a widely available non-invasive optical technique to visualize pressure pulse waves (PWs). Pulse transit time (PTT) is a physiological parameter that is often derived from calculations on ECG and PPG signals and is based on tightly defined characteristics of the PW shape. PPG signals are sensitive to artefacts. Coughing or movement of the subject can affect PW shapes that much that the PWs become unsuitable for further analysis. The aim of this study was to develop an algorithm that automatically and objectively eliminates unsuitable PWs. In order to develop a proper algorithm for eliminating unsuitable PWs, a literature study was conducted. Next, a ‘7Step PW-Filter’ algorithm was developed that applies seven criteria to determine whether a PW matches the characteristics required to allow PTT calculation. To validate whether the ‘7Step PW-Filter’ eliminates only and all unsuitable PWs, its elimination results were compared to the outcome of manual elimination of unsuitable PWs. The ‘7Step PW-Filter’ had a sensitivity of 96.3% and a specificity of 99.3%. The overall accuracy of the ‘7Step PW-Filter’ for detection of unsuitable PWs was 99.3%. Compared to manual elimination, using the ‘7Step PW-Filter’ reduces PW elimination times from hours to minutes and helps to increase the validity, reliability and reproducibility of PTT data.

## Introduction

Photoplethysmography (PPG) is a widely available non-invasive optical technique that uses infrared light and photodiodes to visualize the pressure pulse waves (PWs) in blood vessels by measuring the volumetric changes of pulsating blood and thus the expansion and contraction of the vessels. These PWs result from the contraction of the heart when the blood is pumped through the body [1].

**Fig. 1:**
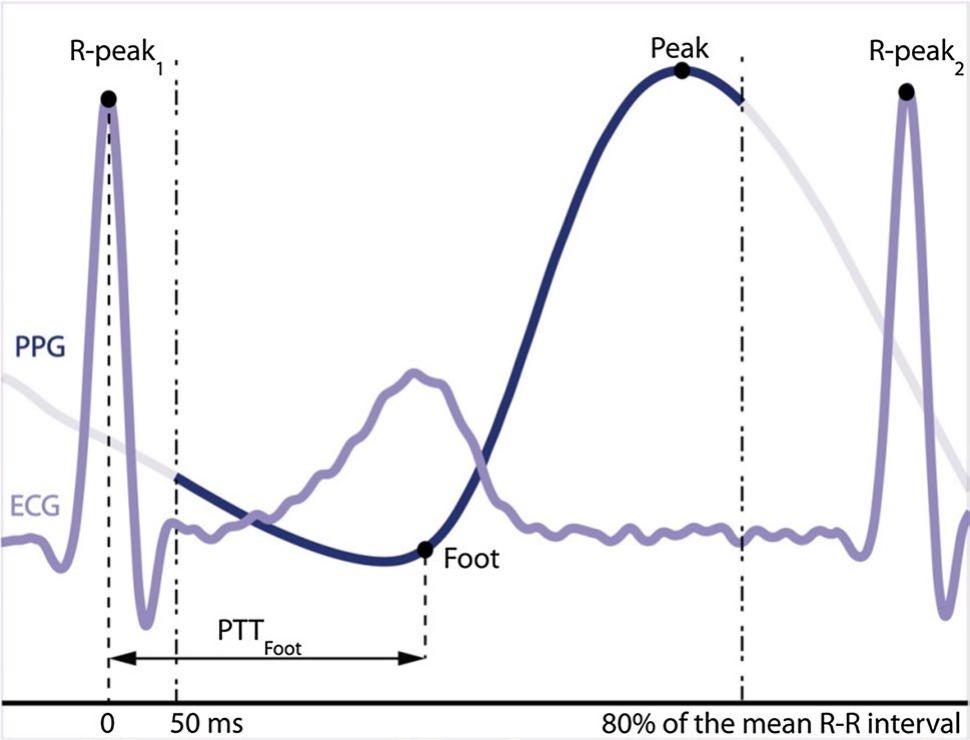
Graphical explanation of PTT calculation when using the PW foot as the landmark that indicates arrival of the PW. *PTT* pulse transit time, *ECG* electrocardiogram, *PPG* photoplethysmography

PPG enables continuous measurement of the PWs [40, 54] and is routinely used in everyday medicine for measuring physiological parameters such as heart rate, blood oxygen saturation (SpO_2_), pulse wave velocity (PWV) and pulse transit time (PTT) [1]. PTT is usually defined as the propagation time of a PW going from the heart to the peripheral arteries and is calculated as the time between the *R*-peak of the ECG and a reference point on the PW measured using PPG (see Fig. 1). PTT is commonly used for assessing arterial stiffness, vessel compliance and sympathetic activity in sleep apnoea patients [43], for measuring endothelial function [36] and as indicator for arterial blood pressure [24]. Generally, PTT is inversely related to PWV [27]. PWV may be considered as the gold standard measure of arterial stiffness [7, 34], which is a very reliable prognostic parameter for cardiovascular diseases [6, 11, 33]. Therefore, PTT is considered to be very useful for studying cardiovascular diseases.

The calculation of PTT is based on tightly defined characteristics of the PW shape. PWs measured using PPG are artefact sensitive to talking, moving, breathing and temperature changes [1]. These artefacts can disturb the shapes of the PWs in such a way that the PWs become unsuitable for further analysis. However, when such unsuitable PWs are nevertheless used for further analysis, nonactual values of calculated parameters may result, which may lead to misinterpretation or even misdiagnosis in clinical practice. Such nonactual values may easily be left unnoticed as these may still fall within the range of commonly encountered values.

Using an algorithm to eliminate unsuitable PWs based on their shape, instead of using, for example, the bandpass filtering method [25] probably gives more reliable results. The bandpass filtering method ignores the fact that a PW within that band is not always suitable, and a PW outside that band is not always unsuitable, which easily results in false positive and false negative filtering results. In addition, the bandpass filtering method does not exclude unsuitable PWs. This paper describes and evaluates an algorithm for assessing the suitability of a PW for PTT analyses based on the reference points detected on the PW.

During PTT measurement in clinical experiments, thousands of PWs may be recorded and may have to be checked manually, as is often done in studies described in literature (see Table 1), which is obviously highly time-consuming. Furthermore, manual assessment of PWs is prone to cause variations due to subjective interpretations.

**Table 1:** Results of the literature review showing how the PTT was determined in the respective studies

References	Population study	Average period	Definition of PTT	Filtering
[32]	Patients	1, 2 min	Foot: maximum of second derivative; 50% is maximum of first derivative; peak: maximum of PW	Algorithm
[20]	Healthy volunteers	The first 50 consecutive PWs	25% peak of PW	Algorithm
[16]	Children	>30 heartbeats	Onset of PW	Algorithm
[15]	Healthy volunteers	1, 5 min	Upstroke of PW	Algorithm
[12]	Children	30 motion-free heartbeats	Upstroke of PW	Algorithm
[14]	Healthy volunteers	50 heartbeats	25% peak point of PW	Algorithm
[18]	Healthy volunteers	8 s	Upslope of PW	Algorithm
[17]	Healthy volunteers	5 min	Slope of PW	Algorithm
[53]	Patients	1 min	Peak of PW	Filtered, not specified
[39]	Healthy blood donors	3, 6 min	Foot, pulse onset of first derivative PW	Filtered, not specified
[60]	Healthy volunteers	30 s, 2 min	Peak of first derivative of PW	Manually
[41]	Healthy volunteers	1 heartbeat	50% peak of PW	Manually
[2]	Healthy volunteers	60 heartbeats	Foot: minimum; peak: maximum	Manually
[3]	Healthy volunteers and patients	100, 400 s	Foot of PW	Median analysis
[35]	Healthy volunteers	1 min	PTTa: foot of PW; PTTb: peak of PW; PTTp: 25% of amplitude of PW; PTTq: max slope of PW	Not specified
[52]	Children	1 min	50% point upstroke of PW	Not specified
[26]	Patients	1 heartbeat	Not specified	Not specified
[55]	Not specified	n/a	Cross-correlation of ECG and derivative PGG	Not specified
[31]	Patients	5 heartbeats, 1 min	Maximal upslope of derivative PW	Not specified
[8]	Patients	1, 5 min	Foot: signal voltage is 10% of baseline value	Not specified
[37]	Healthy volunteers	10 s	Maximum of first derivative	Not specified
[44]	Healthy volunteers	Not specified	Foot/onset of the PW	Not specified
[28]	Healthy volunteers and patients	20–30 s	Foot–foot delay	Not specified
[56]	Healthy volunteers	2, 4 min	Peak of the first derivative of PW	Not specified
[57]	Healthy volunteers	18 s	Foot: onset of PW; PTTdp: max derivative point	Not specified
[46]	Healthy volunteers	1 min	Onset of PW	Not specified
[59]	Healthy volunteers	1 min	Foot of PW	Not specified
[10]	Healthy volunteers	Not specified	50% point on the rising slope of the PPG signal	Not specified
[22]	Children	Not specified	50% point on the rising slope of PW	Not specified
[21]	Children	>30 heartbeats	Upstroke of PW	Not specified
[13]	Healthy volunteers		25% peak of PW	Not specified
[38]	Not specified	Not specified	5% peak systolic value	Not specified
[49]	Healthy volunteers	20 s	Foot of PW	Not specified
[19]	Children	>30 heartbeats	Not specified	Not specified
[48]	Healthy volunteers	15 s	PTT1: peak of second derivative of PW; PTT2: 50% of PW; PTT3: 90% of PW	Not specified
[58]	Healthy volunteers	2 min, 5 min	Foot; maximal sloop; peak	Not specified
[42]	Healthy volunteers	2 min	Upslope of PW	Not specified
[40]	Healthy volunteers	60 heartbeats	Minimum of PW	Not specified
[4]	Patients	2 min	Minimum of PW	Not specified
[25]	Children	1 heartbeat	Onset of PW	PTT outside range of 150 to 400 ms considered invalid
[47]	Healthy volunteers	1 min	Foot, onset of the PW	Visually
[30]	Healthy volunteers and patients	1 min	Foot/onset of PW	Visually
[23]	Healthy volunteers	5 min	Upstroke of PW	Visually
[50]	Healthy volunteers	15 s	Foot of PW	Visually

The studies are sorted by year and grouped by filtering method

The goal of this study was to develop an automated filtering method to quickly and objectively eliminate unsuitable PWs. This algorithm should provide consistent and reproducible results and increase the reliability of PTT values that are calculated based on PW characteristics. Although ECG signal artefacts may also cause nonactual PTT values, this article focuses on the PW shape.

## Materials and methods

### Literature study

In order to develop a proper algorithm for eliminating unsuitable PWs, it should first be clear how the suitability of a PW for PTT calculation should be defined. In literature, the foot, minimum value, point of steepest ascend, or peak or maximum value of a pulse wave are the locations on the PW that are commonly used to calculate the PTT. These locations are all used under the assumption that every PW has a certain characteristic shape and that the arrival of such a location on the PW indicates the arrival of the PW. In order to enable automated extraction of such data, it should be well defined where a PW starts and how the characteristic shape of this wave should be described.

For PTT calculations, there are several definitions of PTT, each of which using a different location on a PW as the reference point indicating the arrival of the PW. A literature study was done to get an overview of methods that are being used to calculate the PTT in clinical experiments and to see if and how unsuitable PWs are being eliminated. The literature study was conducted in PubMed for studies up to 25 June 2015 and using the search query: ‘*photoplethysmography’* [*MeSH Terms*] *OR* (‘*photoplethysmography’* [*All Fields*]) *AND ‘pulse transit time’* [*All Fields*] *AND* (‘*humans’* [*MeSH Terms*] *AND English*[*lang*]) *NOT Review*[*ptyp*]. The query returned 53 studies, of which the relevant ones are listed in Table 1. Nine studies were eliminated because PTT was measured using a different technique than PPG or because the study focused on monitoring devices. Four characteristics of the studies are extracted and listed in Table 1:

- Population;
- Period over which PTT was averaged;
- Definition of PTT used;
- Method of filtering applied to the data.

#### Population

The populations described in the studies listed in Table 1 consisted of healthy volunteers in 26 studies, of a combination of healthy volunteers and patients in three studies and of only patients in six studies. In seven studies the population consisted of children, and in two studies the population was not specified.

#### Period over which PTT was averaged

In many studies the PTT values used as the outcome measure were not single-PW PTT values of all individual PWs, but were average PTT values over a certain number of heartbeats or a certain period of time. These averaging periods ranged from 5 heartbeats to 6 min. An averaging period of 1 min was most common (10×). The smaller the number of PTT values included in the averaging period, the more sensitive the calculated value will be to unsuitable PWs. Yet, in patients having many unsuitable PWs, even PTT values obtained from long averaging periods may be highly affected by unsuitable PWs.

#### Definition of PTT used

The PW foot is the reference point most commonly used (19 studies) in PTT analysis to pinpoint the arrival instance of a PW. However, the PW foot was not always defined identically. In three studies the foot was defined as the minimum of the PW, and in three studies the foot was defined as the maximum of the second derivative of the PW. In seven studies the ‘onset’ of the PW was defined as its foot and six studies did not define what was used as the foot of a PW. In four studies the PW peak was taken as the PW arrival instance, while in eight studies the maximum upslope of the PW was used.

#### Method of filtering applied to the data

Only seven reports mentioned that unsuitable PWs were eliminated manually or visually, but the criteria were not defined clearly or were not mentioned at all. In the majority of the studies it was unclear whether or not any PWs were eliminated. Some reports mentioned the use of a PW elimination algorithm, but did not specify the applied algorithm. Gil et al. [25] used a filter that eliminated any PTT values below 150 or above 400 ms before further analysis. Although this filter may eliminate PWs that are so heavily distorted that the calculated PTT becomes unrealistically low or high, it does not remove any nonactual values that are within normal ranges and it may eliminate valid values that simply are unusually low or high.

### PW elimination algorithm

**Fig. 2:**
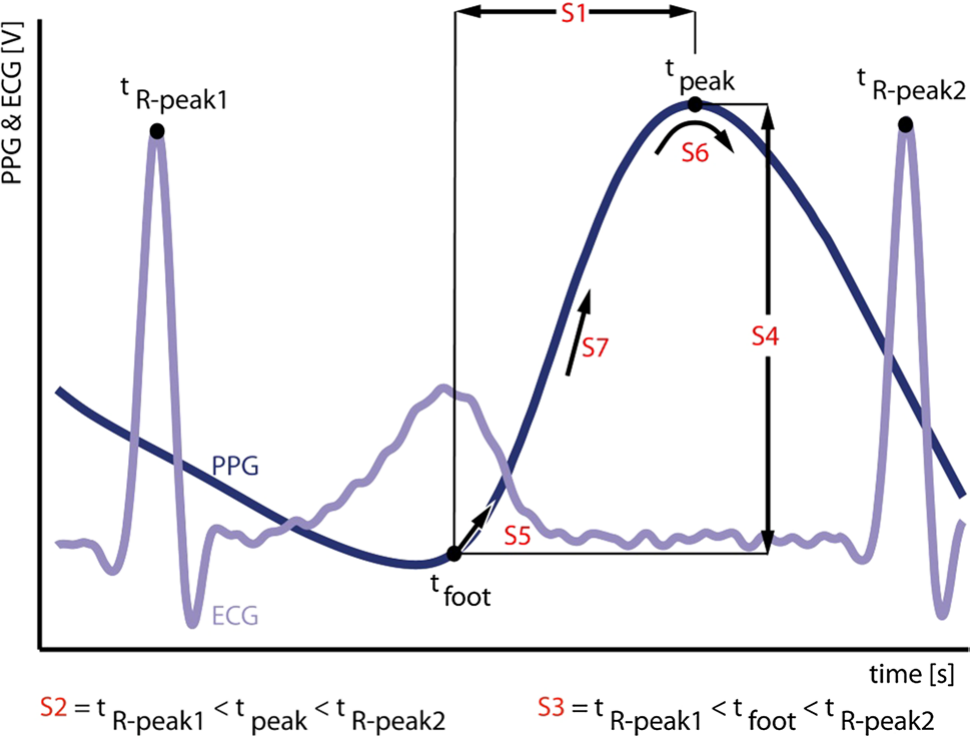
Graphical representation of the seven PW elimination criteria of the ‘7Step PW-Filter’. *ECG* electrocardiogram, *PPG* photoplethysmography. *S1* the detected PPG_foot_ should precede the detected PPG_peak_ in time, *S2* the detected PPG_peak_ should be in the same heartbeat as the ECG *R-*peak, *S3* the detected PPG_foot_ should be in the same heartbeat as the ECG *R*-peak, *S4* the detected PPG_foot_ should have a lower magnitude than the detected PPG _peak_, *S5* the detected PPG_foot_ must be in an upward slope of the valley of the PW, *S6* the PW should be complete, the detected PPG_peak_ should be at a convex maximum of the PW, *S7* the steepest rising part of the PW (maximum of its first derivative) must be situated between the detected PPG_foot_ and the detected PPG_peak_

In 1937 Hertzman and Spealman [29] were the first to describe the shape of a PW, dividing PWs in two phases: the anacrotic phase consisting of the rising slope of the PW and the catacrotic phase consisting of the falling slope of the PW. In the catacrotic phase a dicrotic notch is usually seen in subjects with healthy compliant arteries [1]. Common physiological parameters, such as PW amplitude, PTT and PWV, are generally calculated based on the assumption that a PW has the described shape. Based on these principles, a PW elimination algorithm was formulated that checks whether a PW matches the characteristics of the described predefined shape. The algorithm exists of a list of seven criteria that a PW has to meet to be deemed a suitable PW (see also Fig. 2):

- S1: The detected PPG_foot_ should precede the detected PPG_peak_ in time.
  - *t*_foot_ < *t*_peak_
- S2: The detected PPG_peak_ should be in the same heartbeat as the ECG *R*-peak
  - *t*_*R*-peak1_ < *t*_peak_ < *t*_*R*-peak2_
- S3: The detected PPG_foot_ should be in the same heartbeat as the ECG *R*-peak
  - *t*_*R*-peak1_ < *t*_foot_ < *t*_*R*-peak2_
- S4: The detected PPG_foot_ should have a lower magnitude than the detected PPG_peak_
  - PPG_peak_–PPG_foot_ > 0
- S5: The detected PPG_foot_ must be in an upward slope of the valley of the PW
  - First derivative at PPG_foot_ > 0
- S6: The PW should be complete; the detected PPG_peak_ should be at a convex maximum of the PW
  - Second derivative at PPG_peak_ < 0
- S7: The steepest rising part of the PW (maximum of its first derivative) must be situated between the detected PPG_foot_ and the detected PPG_peak_
  - *t*_foot_ < *t* maximum of the first derivative < *t*_peak_

If one or more of the criteria are not met by the PW, that PW is deemed unsuitable and will be eliminated. This PW elimination algorithm will further be referred to as the ‘7Step PW-Filter’.

### PW analysis

**Fig. 3:**
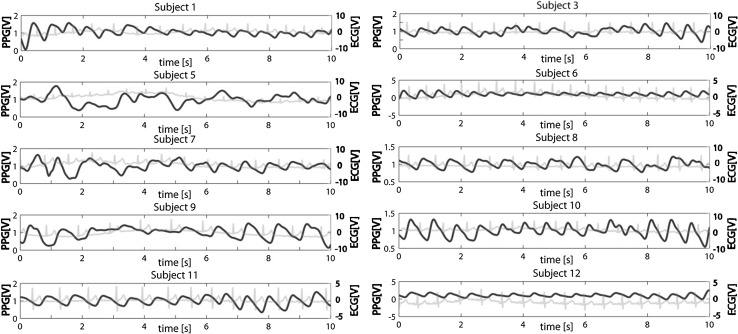
Examples of PPG signals and their corresponding ECG signal for all ten subjects. Each example shows the first 10 s of the baseline measurements on volunteers under tranquil conditions. Subjects 6 and 12 have a PPG signal without any noticeable artefacts. Subjects 5 and 9 have many artefacts, rendering it difficult to identify the suitable PWs

The ‘7Step PW-Filter’ was validated using a dataset consisting of PWs that were collected from the first ten healthy volunteers (seven male, three female, ages between 23 and 25 years) from a previous study conducted by the authors (medical ethics committee approval report MEC-2012-489, Erasmus MC, Rotterdam, the Netherlands) [51]. In this study, the PTT was measured using two PPG sensors, one on each index finger (TSD200 with the PPG100C amplifier, Biopac Systems, Inc, USA) and three external ECG leads (ECG100C amplifier, Biopac Systems, Inc, USA). The three ECG leads were placed on the subject’s right ankle and both wrists. The subject sat in a comfortable position under tranquil conditions and was instructed not to talk or move during the measurement. The PWs were measured for 180 or 300 s in each subject. The subjects received three painful, heat-induced stimuli during the measurements. To give a general impression of the data, Fig. 3 shows 10 s of the datasets of all subjects. The figures clearly illustrate that it is not always easy to recognize the PWs and their ends or beginnings.

The system used for measuring PTT registered the subject’s ECG and PPG signals, which were simultaneously converted to digital signals using AcqKnowledge v3.7.3 software (Biopac Systems, Inc, USA) at a sampling frequency of 2 kHz. Matlab R2010a (The MathWorks, Inc) was used for the data analysis. The PPG signals were filtered with a fourth-order low-pass Butterworth filter with a cut-off frequency of 9 Hz. The PTT was determined by calculating the time between the *R-*peak of the ECG (*t*_ECG *R*-peak_(*n*)) and the foot of the PW (*t*_PPG foot_(*n*)):

1 $$\text{PTT}(n) = t_{{\text{PPG}_{{\text{foot}}} }} (n) - t_{{\text{ECG}_{{R - \text{peak}}} }} (n)$$

where *n* is the sequence number of the heartbeats. The *R-*peaks in the ECG were found using an off-the-shelf Matlab function called ‘*R*-peakdetect’ [9]. In order to always use an *R-*peak and PW that belonged to the same heartbeat, the PWs were digitally clipped from 50 ms after the occurrence of the *R-*peak to 80% of the average interval between two *R-*peaks (see Fig. 1). The peak of a PW was determined as the maximum of the PW and was found using an off-the-shelf Matlab function called ‘Peakdet’ [5]. The foot of a PW was located at the maximum of the second derivative of that PW.

**Fig. 4:**
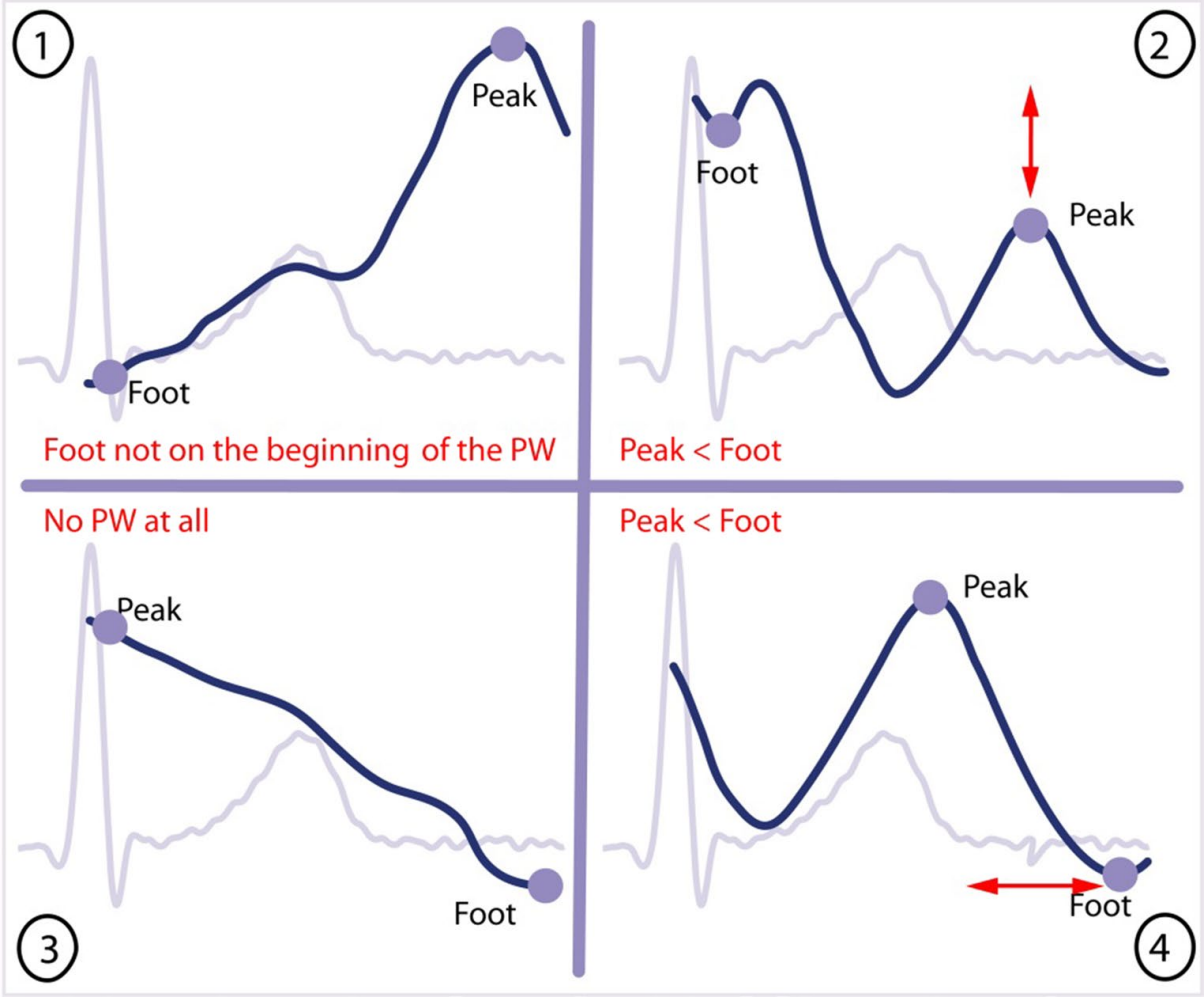
Examples of unsuitable PWs. Section *1* shows a detected PW foot that is not on the beginning of the PW but at the beginning of the clipped dataset. This is not correct because the foot has to be at the start of the PW itself and not at the start of the clipped dataset. Section *2* shows a detected PW peak being lower than the detected foot of the same PW. This is not correct because the peak should always be higher than the foot. Section *3* shows a PW that is nonrecognizable at all, causing the foot and peak to be incorrectly placed at the extreme values the of the clipped dataset. Section *4* shows a detected PW foot occurring later than the detected peak of the same PW. This is not correct, because the foot of the PW has to precede the peak of the PW

Figure 4 shows four examples of unsuitable PWs in which the Matlab detection software placed the foot and/or peak on a wrong location. Figure 4.1 shows a detected PW foot that is not on the beginning of the PW but at the beginning of the clipped dataset. This is not correct because the foot has to be at the start of the PW itself and not at the start of the clipped dataset. Figure 4.2 shows a detected PW peak being lower than the detected foot of the same PW. This is not correct because the peak should always be higher than the foot. Figure 4.3 shows a PW that is not recognizable at all, causing the foot and peak to be incorrectly placed at the extreme values the of the clipped dataset. Figure 4.4 shows a detected PW foot occurring later than the detected peak of the same PW. This is not correct, because the foot of the PW has to precede the peak of the PW.

### Validation

To verify whether the ‘7Step PW-Filter’ eliminates only and all of the unsuitable PWs, all PWs of the obtained dataset were put through the ‘7Step PW-Filter’ as well as visually checked by the first author (M.H.N.V.) and manually marked for elimination if deemed unsuitable (referred to as ‘manual elimination’ from now on). The literature review (Table 1) showed that manual/visual filtering was the most common way of filtering. During manual selection of unsuitable PWs in the current study, it was visually judged whether the detection software in Matlab placed the points of interest on the correct locations on the PWs. If any of those points was judged to be placed wrongly, the PW was marked for elimination.

To analyse the performance of the ‘7Step PW-Filter’ compared to the manual elimination, the outcome of the two methods was considered as a binary classification test. Their sensitivity and specificity were used as statistical measures of performance. For each PW eliminated by the ‘7Step PW-Filter’ the reason for elimination was recorded by registering which of the seven criteria were not met.

To explore the effect of eliminating unsuitable PWs before averaging a calculated outcome variable over a certain period, the mean PTT was calculated after using several distinct averaging periods for subject number five and compared for three filtering situations: no filtering, filtered by the ‘7Step PW-Filter’ and filtered by manual elimination. This comparison was done for means of the entire dataset in which the individual PTT values were calculated as averages per 60, 30 and 5 heartbeats or taken for each individual heartbeat.

To gain insight into the effect of applying a filter on the PTT values instead of applying the elimination algorithm on the actual PWs, the filter of Gil et al. (eliminating any PTT values below 150 or above 400 ms) was applied to the dataset of subject number five. Consecutively, it was checked to what extent the Gil method and the 7Step PW-Filter included and excluded the same data points.

All PTT analyses were done using an Intel Core i7-2640 M CPU 2.80 GHz, 64-bit operating system with Windows 7 professional Service Pack 1, Microsoft Corporation, Redmond, WA, USA.

### Statistical analysis

Three performance measures of the ‘7Step PW-Filter’ were calculated using the manual elimination results as a reference: the sensitivity, the specificity and the overall accuracy. In the context of the current work, the sensitivity is a measure of the ‘7Step PW-Filter’ ability to eliminate unsuitable PWs in accordance with the manual elimination. The specificity is a measure of the ‘7Step PW-Filter’ ability to keep in suitable PWs in accordance with the manual elimination. The overall accuracy was calculated as the total of the number of true positive PWs plus the number of true negative PWs, divided by the total number of PWs. These three performance measures should ideally be close to 100% under the assumption that the manual elimination results are valid and reliable. The analysis was performed using SPSS version 20.0 (SPSS, Inc., Chicago, IL, USA) and Matlab R2010a (The MathWorks, Inc). The limit for statistical significance was chosen as *p* < 0.01.

## Results

The complete dataset consisted of a total of 7746 PWs, obtained from 10 subjects. Manual elimination eliminated 164 PWs (2.1%), based on visual inspection of the PWs. The ‘7Step PW-Filter’ eliminated 209 PWs (2.7%), based on the list of seven criteria. Full processing of the 7746 PWs took about 5 h for manual elimination and under 5 min for the ‘7Step PW-Filter’. The manual elimination and ‘7Step PW-Filter’ agreed on the elimination of 158 out of all eliminated PWs (which is 2.0% of the total number of PWs, 96% of the manually eliminated PWs and 76% of the PWs eliminated by the ‘7Step PW-Filter’).

Additionally, six PWs were manually eliminated while not having been eliminated by the ‘7Step PW-Filter’. These six PWs were manually eliminated because there was no visually recognizable beginning of the PW.

Furthermore, 51 PWs were eliminated by the ‘7Step PW-Filter’ while not having been eliminated manually. Mostly (in 39 instances), these PWs did not meet Criterion S5 (the detected PPG_foot_ must be in an upward slope of the valley of the PW). In eight instances the PW did not meet Criterion S2 (the detected PPG_peak_ should be in the same heartbeat as the ECG *R*-peak). In one instance the ‘7Step PW-Filter’ eliminated a PW on Criterion S7 (the steepest rising part of the PW should be between the detected PPG_foot_ and the detected PPG_peak_). One PW was eliminated on Criteria S3 and S5, and two PWs were eliminated on Criteria S5 and S7.

The ‘7Step PW-Filter’ had a sensitivity of 96.3% and a specificity of 99.3%. The overall accuracy of the ‘7Step PW-Filter’ was 99.3% (Table 2).

**Table 2:** Sensitivity and specificity of the manual elimination and the 7Step PW-filter elimination

	‘7Step PW-Filter’ outcome positive	‘7Step PW-Filter’ outcome negative
Manual elimination outcome positive	2.0%–158 PWs	0.1%–6 PWs
Manual elimination outcome negative	0.7%–51 PWs	97.2%–7531 PWs

	Sensitivity	Specificity
	96.3%	99.3%

‘Positive’ indicates that a PW was marked as unsuitable and eliminated. ‘Negative’ indicates that a PW was marked as suitable and kept in the dataset

In the dataset of subject number five the ‘7Step PW-Filter’ eliminated 125 PWs, which was 16.4% of the total dataset. Manual elimination resulted in 101 eliminated PWs, which was 13.3% of the total dataset.

**Fig. 5:**
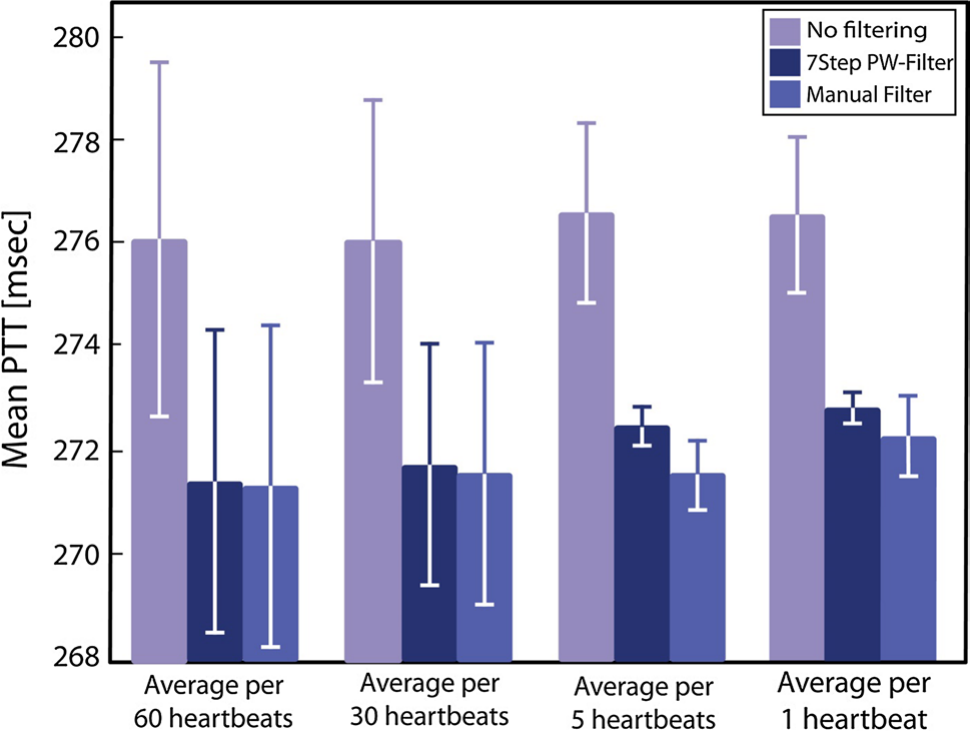
Mean PTT values of the dataset of subject number five; no filtering (‘*no filtering*’), after manual elimination of unsuitable PWs by the first author (‘*manual filter*’) and after applying the ‘7Step PW-Filter’ (‘*7Step PW-Filter*’). The given PTT values are means over the entire dataset in which the individual PTT values were taken as averages per 60, 30 and 5 heartbeats or taken for each individual heartbeat. The *whiskers* indicate the standard errors of the means

Figure 5 shows the effect of eliminating unsuitable PWs before averaging a calculated outcome variable over a certain period. The difference between using filtered and unfiltered PPG data can lead to a difference in the calculated average PTT of up to 5 ms, which is 1.8% of the original outcome value. The difference between the two filtering methods was less than 0.9 ms. Eliminating unsuitable PWs was over 60 times faster when using the ‘7Step PW-Filter’ (under 5 min) than when doing manual elimination (about 5 h).

**Fig. 6:**
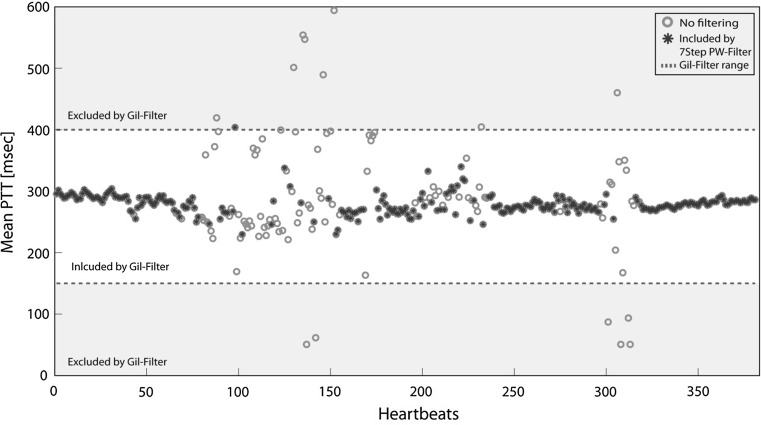
Comparison of the ‘7Step PW-Filter’ with the Gil method. The *figure* shows PTT values for the dataset without no filtering (‘*no filtering*’), the boundaries set by the Gil method (‘*Gil filter range*’) and the PTT values remaining in the dataset after eliminating unsuitable PWs with the ‘7Step PW-Filter’ (‘*7Step PW-Filter*’)

Figure 6 shows the difference between using unfiltered data, using the Gil method and using the ‘7Step PW-Filter’. The mean PTT over the entire dataset was comparable for all three methods (no filter: 282 ms, Gil method: 281 ms, 7Step filter: 279 ms). However, the results clearly show that although few suitable PWs fell outside the Gil range, a very large number (91) of unsuitable PWs were included in the analysis when using the Gil method.

## Discussion

The literature study revealed that filtering techniques that are used to eliminate unsuitable PWs are often not described and differ between studies. In fact, most studies do not report whether any or what kind of filtering algorithm was used to eliminate unsuitable PWs. Some studies report using manual techniques to select unsuitable PWs, but these techniques are labour intensive, subjective and often not fully described either.

By using seven morphologic criteria to determine the suitability of PWs for PTT analyses, the ‘7Step PW-Filter’ eliminated 158 out of the 164 PWs that were also eliminated by the manual method. The six PWs not eliminated by the ‘7Step PW-Filter’ were eliminated manually because a clear onset of the uprising slope could not be found visually in these six PWs. The advantage of the ‘7Step PW-Filter’ is that it objectively determines this onset and determines whether its location fits the characteristics of a suitable PW. Of the 51 PWs that were eliminated by ‘7Step PW-Filter’ while not being eliminated by the manual method 39 PWs were eliminated because the maximum of the second derivative of the PW was not situated on an upward slope. This suggests that visual inspection of the PWs is less reliable because the location of a maximum of a second derivative is very hard to pinpoint by eyeballing.

In order to show the relevance of eliminating unsuitable PWs, a case study on PTT data from a volunteers study was conducted. In that study the mean PTT values were to be calculated based on finding specific landmarks on PWs and ECG data. The case study showed that when reporting a mean PTT of a dataset the effect of using or not using elimination of unsuitable PWs can be considerable. PTT values dropped by 1.5–1.8% when applying either manual elimination or the ‘7Step PW-Filter’ as compared to using unfiltered data. Whether the mean PTT was determined for a range of PTT values derived from short (5 heartbeats) or long (60 heartbeats) averaging intervals had little effect on the mean PTT.

However, the smaller the number of PWs over which the PTT values were averaged, the more sensitive the calculated mean PTT was to unsuitable PWs. As fast physiological responses or fluctuations can only be measured or monitored properly without averaging over too many PWs, effective and reliable PW elimination algorithms are quintessential for obtaining reliable measurement of fast physiological responses. When measuring changes in PTT, significant results reported in the literature that are deemed clinically relevant amount about 10–20 ms [4], which is a change of 3–7% with respect to a common baseline PTT of 300 ms, implying that unremoved unsuitable PWs could potentially account for 50% of such results. This clearly indicates that it is essential to dispose of unsuitable PWs, as these can have a considerable effect on clinically relevant outcome values.

As PTT is defined as the time difference between the ECG *R*-peak heartbeat and a reference point on the PPG signal of the corresponding PW, having proper ECG waves is just as relevant as having suitable PWs. However, several studies have already shown the robustness of various methods for detecting ECG *R*-peaks [45]. Therefore, this study focused solely on the PPG signal.

Visually selecting unsuitable PWs and removing these manually is highly time-consuming. It took about 5 h to process the data of only ten subjects. Additionally, the manual elimination may be quite subjective. Although the manual elimination was taken as gold standard, it should be noted that the manual filtering may give varying results, depending on who conducts the filtering. However, in the current study the manual filtering was done by an expert researcher to as much as possible avoid bias in favour of the algorithm, Consequently, for less experienced researchers, large datasets and PWs hard to judge visually, the 7Step PW-Filter’ potentially offers even larger benefits. The ‘7Step PW-Filter’ offers great time savings compared to manual elimination and can be implemented in many coding languages due to its simple and straightforward concept.

Gil et al. [25] used a filter that eliminated any PTT values that were below 150 or above 400 ms, before further analysis of the PTT data. However, if the shape of a PW does not show the characteristics that allow calculating a PTT but the PTT value is calculated anyway, this may result in PTT values that fall within the Gil criteria but are still nonactual data. The comparison between the ‘7Step PW-Filter’ and the Gil method confirmed that although the calculated outcome value (mean PTT in this case) may be only slightly affected by the filtering method used, the Gil method kept a very large number of nonactual PTT values in the dataset. The ‘7Step PW-Filter’ did remove all unsuitable PWs, thereby preventing nonactual PTT values from polluting the filtered dataset. Therefore, the ‘7Step PW-Filter’ should be preferred.

’The ‘7Step PW-Filter’ was validated on healthy volunteers only and with potential sources of motion artefacts in the PWs being avoided. This clearly is quite an ideal situation. In clinical practice, patients may have cardiovascular disease, which affects arterial stiffness and could affect the shape of the PWs. Furthermore, patients may be anxious, in pain, coughing or moving for other reasons, which may also affect and most likely deteriorate the shapes of the PWs. In such situations, experience has shown that the number of unsuitable PWs increases, which makes proper filtering even more important. An extensive quantification of the performance of the ‘7Step PW-Filter’ in such situations has yet to be conducted.

Apart from the artefacts caused by talking and moving, the shapes of the PWs can also be affected by too high contact forces between the subject and the sensor, as was reported by Teng and Zhang [48, 50] when using reflective PPG sensors. This effect was also noticed during the current study: when the PPG sensors were strapped too tightly to the fingers, the blood flow stagnated, causing the PWs to deteriorate both in amplitude and in shape. Therefore, care should be taken to limit or avoid any contact forces when using PPG sensors.

Although this study focused on applying PW elimination for PTT calculations, the advantages of automated elimination of unsuitable PWs will also apply when aiming at other outcome parameters, such as PW amplitude, heart rate, SpO_2_, blood pressure, cardiac output and PWV. For such cases the list of criteria in the ‘7Step PW-Filter’ may have to be adapted.

## Conclusion

In order to obtain valid PTT data from PPG measurements, it is quintessential to only use PWs that contain the morphological landmarks on which the definition of PTT is based. The comparison of data analysis and filtering methods in this study showed that without filtering, the period over which PTT values are averaged can strongly affect the calculated outcome values. Using unfiltered data may result in deviations in the calculated PTT values that are close to the orders of magnitude of commonly measured effect sizes in patient and healthy volunteer studies. Compared to manual elimination, using the ‘7Step PW-Filter’ reduces PW elimination times from hours to minutes and helps to increase the validity, reliability and reproducibility of PTT data.
